# An Innate Despair: The Philosophical Limitations of Transhumanism and its Misplaced Hope in Human Enhancement

**DOI:** 10.1177/00243639241281977

**Published:** 2024-10-07

**Authors:** Paschal M. Corby

**Affiliations:** 1 97581 University of Notre Dame Australia (Sydney Campus), Chippendale, NSW, Australia

**Keywords:** anthropology, despair, enhancement, hope, philosophical positivism, transhumanism

## Abstract

This paper sets forth some philosophical foundations of the transhumanist project, drawing on its roots in philosophical positivism and its confidence in the liberating power of technology. Such confidence is interpreted within the sphere of hope, departing from transhumanism's negative presumptions regarding the human condition, and embracing its aspirations for a humanity without limits. However, it is the claim of this paper that such hope is deceptive. Since transhumanism is incapable of grasping the depths of the human person, limited by its own philosophical categories, it both underestimates the human capacity for interior transformation and misunderstands the real nature of hope. Thus, transhumanism is marked by a deeply rooted despair that wears the mask of an insufficient hope.

## Introduction

Much has been written about transhumanism over the past twenty years, such that it is difficult to add anything new to the debate. This present paper does not presume to be novel in this regard. Instead, its main purpose is to draw out the philosophical foundations of the transhumanist project, setting forth its roots in philosophical positivism and its confidence in the liberating power of technology, and to consider the implications for anthropology, the determination of human purpose, and object of human hope. In this regard, it may provide a platform to critique other contemporary prospects of “enhancement” that share transhumanist tendencies, such as artificial intelligence and Neuralink, gender fluidity, as well as attempts at hybridization through transgenesis or xenotransplantation. While this paper chooses to focus specifically on transhumanism, the limitations exposed could equally be applied to these other proposals that threaten to profoundly change humanity, compromise its uniqueness, and mutate the nature of human hope.

## Why Transhumanism?

In the introduction to their co-edited work, *Human Enhancement*, Julian Savulescu and Nick Bostrom, both adherents to the transhumanist project, ask a fundamental question: “Are we good enough?” Presuming a negative response, they immediately follow with another: “If not, how may we improve ourselves?” (Savulescu and Bostrom 2009, 1). As the narrative unfolds, it appears that humans are not good enough, and there is reason—indeed, a moral duty—to enhance ourselves through the ingenuity of science and technology. At the root of this imperative for enhancement is a profound sense of despondency and frustration with our human condition. In this, transhumanists clearly respond to a valid human experience. Discontentment and disappointment are part of every life. Before our limitations, we all yearn for something more, desire to go further, strive to improve ourselves in one way or another. The human spirit has always striven towards self-improvement, and the historical advances in human culture would be unthinkable without this drive towards enhancement. The real question, however, is whether transhumanists like Savulescu and Bostrom interpret the experience of human dissatisfaction correctly, and whether their confidence in the methods of human enhancement, especially through biotechnology, corresponds to genuine human hope or masks an underlying despair.

Transhumanism can be defined as a project of enhancement through the application of biotechnology to move human beings beyond their natural limitations: to make them smarter and stronger; to assist them to be happier and morally responsible; and/or to enable them to live longer. The novelty of the transhumanist project does not lie in the biotechnologies that it advances. Many of the technologies proposed are already on-line and used therapeutically. Pharmaceuticals, genetic manipulation, nanotechnology, and some brain–computer interfaces (e.g., cochlear implants for the hearing impaired) already exist in clinical practice and promise further advances to treat and cure disability and disease. The difference lies in transhumanism's desire to engage such technologies toward ends surpassing the therapeutic. Transhumanists seek to enhance the human condition beyond any notion of “normal” functioning (which, as shall be seen below, they fundamentally deny); to make us, in the words of Michael Sandel, “better than well” (Sandel 2009, 71). In so doing, they purposefully disregard the therapy-enhancement distinction with its presumption of normative function and render any improvement as an enhancement, that is, an “improvement” of a person's life relative to their former condition.

From this perspective, the prospects for enhancement are limitless. According to the *Transhumanist Declaration*, first drafted in 1988, humanity's potential is still mostly unrealized. Signatories of this declaration envision the possibility of harnessing the human potential to transcend its current form by overcoming aging, cognitive shortcomings, physical limitations, and suffering. To this end, they favor morphological freedom—the right to modify and enhance one's body, cognition, and emotions. This freedom includes the right to use techniques and technologies to extend life; preserve the self through cryonics, uploading, and other means; and to choose further modifications and enhancements.

The term “transhumanism” was first used in 1957 by Julian Huxley in his book *New Bottles for New Wine*. Huxley envisaged a transhuman state at the end of an evolutionary process guided by science and technology. He conceives of “man remaining man, but transcending himself, by realizing new possibilities of and for his human nature” (Huxley 1957, 17). Huxley envisions humanity as the final frontier for exploration.

We have pretty well finished the geographical exploration of the earth; we have pushed the scientific exploration of nature, both lifeless and living, to a point at which its main outlines have become clear; but the exploration of human nature and its possibilities has scarcely begun. A vast New World of uncharted possibilities awaits its Columbus (Huxley 1957, 14).

From these initial aspirations, contemporary proponents of the transhumanist cause, encouraged by advances in science and technology, are confident of its realization. But in doing so, they refine Huxley's end, making a distinction between a *transhuman* and a *posthuman* state. The distinction between the two is not fixed. Generally speaking, the *transhuman* (or “transitional” human) is one whose physical, intellectual, and psychological capacities are enhanced with respect to the present human experience, but not to the point of creating a new species. The *posthuman*, on the other hand, refers to a radically new state characterized by an amplification of one's capacities that would constitute a new kind of being.

In seeking to enhance humanity beyond its natural limitations, the transhumanist project embraces a wide range of proposals, from simple and achievable measures to the scientifically technical and bizarre. It moves between scientific fact and science fiction. In light of its more futuristic aspirations, transhumanism might not seem to warrant serious reflection. Before the presence of real, existent bioethical dilemmas such as new threats to life, the unjust distribution of healthcare resources, and the perennial issues surrounding life at its beginning and end, devoting time to an improbable possibility might appear to be a distraction or a luxury that we can ill afford. Yet the prospect of transhumanism has caught the imagination of not just a few contemporary bioethicists and philosophers, inspiring centers of research, and sparking a lively debate between its supporters and critics. And this interest seems to only increase. In a short space of time, transhumanist ideas have moved from the fringes of philosophical inquiry into the mainstream.

However, beyond the demands of public interest, the transhumanist proposal demands a response since it touches on the very meaning of human life and hope. In asking why one should take seriously the proposals of transhumanists, American bioethicist Leon Kass writes:

It raises the weightiest questions of bioethics, touching on the ends and goals of the biomedical enterprise, the nature and meaning of human flourishing, and the intrinsic threat of dehumanization (or the promise of superhumanization). It compels attention to what it means to *be* a human being and to be active *as* a human being. (Kass 2003, 10)

In their pursuit to enhance and even transcend the current human condition, transhumanists cast shadows over the goodness of life while raising hopes for a bright and better future. Thus, even if many of its imaginative prospects do not eventuate, it demands a reflective response, for our hopes and aspirations define who we are. “What we hope for tells us a great deal about who we are,” says Gilbert Meilaender (2013, 23). Even if the quest for transcending our humanity turns out in the end to be no more than a “pipe dream,” the fact that it is even proposed is significant.

## The Object of Transhumanist Hope and its Philosophical Foundations

Encouraged by the possibilities of technology, the transhumanist cause is characteristically hopeful. However, the nature of such hope is of a particular kind. In essence it is an immanent, this-worldly hope: what may be described as a “future-oriented ideology” (Burdett and Lorrimar 2019, 243) that aspires to a “brave new world” (*à la* Julian Huxley's more famous brother, Aldous). That which was once confided in God or fate (for the relief of suffering and hope of prospering) is now firmly placed within the sphere of the human capacity to know and act, and increasingly finds its solace in a rationality that is the product of technical science. This immanentization of hope is rooted in a deeper philosophical ideology, drawing specifically on the Enlightenment's confidence that human enhancement comes by way of rational knowledge alone, with knowledge itself reduced to positive categories of the verifiable and demonstrable, especially in the fields of science, mathematics, and history (Ratzinger 2004, 61).

In the process, however, critics point out that the object of rationalistic hope is narrowed and the significance of man diminished. As Joseph Ratzinger writes:

The criterion of rationality is taken exclusively from the experience of technological production based on science. Rationality is oriented to functionality, to effectiveness, and to an increase in the quality of life for all. This entails a use—indeed a domination—of nature that is problematic in view of the dramatic environmental problems our world now faces. But man's domination of his own self nonchalantly takes ever greater steps toward the realization of Aldous Huxley's vision. Man is no longer to be born in an irrational manner but is to be produced rationally. Man as a product is subject to the control of man. Imperfect individuals must be weeded out; the path of planning and production must aim at the perfect man. Suffering must disappear, and life is to consist of pleasure alone. (Ratzinger 2006, 157)

In Ratzinger's analysis, the reduction of hope to rational, technical science has immediate and negative consequences for anthropology. The relevance of his analysis to the transhumanist cause seems quite clear, with its projection of hope onto biotechnology and its quest to re-create human beings.

## The Anthropological Question

The nature of transhumanist hope is thus rooted in its materialistic and evolutionary anthropology. Emerging from its roots in Enlightenment rationalism, and heir to an evolutionary philosophy, transhumanism holds to the conviction that human beings in their current form are incomplete but improvable; “a work-in-progress, a half-baked being that we can learn to remold in desirable ways” (Bostrom 2003, 493). They consider human enhancement to be in continuity with evolution, either by giving it a helping hand or by correcting its shortcomings, to move human beings further along evolution's trajectory by replacing natural selection with technological enhancement.

However, while transhumanism finds its rationale in the evolutionary process, it assumes a novel stance in relation to the end of evolution, and its concept of the human person is somewhat removed from the humanist anthropology which originally guided evolutionary theory. The difference lies in the human person's relation to, or distinction from, Nature. Italian philosopher Maurizio Faggioni (2009, 406–7) explains that while classical evolutionary theory regards human beings as “the point of arrival and the apex of an ascending movement,” transhumanists have purged evolution of its anthropocentrism and has robbed humanity of any special significance.

In the classical evolutionary interpretation, the human person emerges as distinct from the world. He or she is gradually “purified” and separated from Nature, “*eliminating the animal and the machine from his image*” (Tintino 2014, 388). In contrast, the prospect of transhumanism, especially in its posthuman aspirations through the hybridization of human and non-human animals, or of person and machine, is leading towards a new *alterity*—a diffusion of human distinctness and a blurring of boundaries (Pepperell 2005, 34). The transhuman, therefore, does not exist simply as a hyper-technologized humanity. Rather, it results from the “elimination” and “fluidization” of differences, and “the annihilation of all the boundaries that make ‘human’ a human being” (Valera 2014, 483).

Because there is no ultimate reason why human beings should have evolved as they did, nothing special or final about their current form, then there is no reason why it should remain as it is. According to Max More: “Transhumanists regard human nature not as an end in itself, not as perfect, and not as having any claim on our allegiance. Rather, it is just one point along an evolutionary pathway and we can learn to reshape our own nature in ways we deem desirable and valuable” (More 2013, 4). Holding no claim on our allegiance, there is no moral imperative to continue as we are, no “principled reasons to remain human if we can create creatures, or evolve into creatures, fundamentally ‘better’ than ourselves;” no objection to enhancements that “would alter or destroy human nature” (Buchanan 2011, 138). Transhumanist author John Harris likens the attempt to preserve our current human nature to “the absurdity of our common ape ancestors in Africa getting together with a simian agenda to block evolution so that simian nature would be preserved as ‘the common heritage of simian kind’” (Harris 2010, 40).

This denial of human distinction, and the elimination of boundaries, ultimately amounts to “the negation of man” (Valera and Tambone 2014, 362) or of what C. S. Lewis prophetically referred to as his *abolition* (Lewis 2009, 65) of the human person treated as an artefact, as a mere “natural object,” or “as raw material for scientific manipulation to alter at will” (Lewis 2009, 73). Lewis warned against this end, troubled by the prospect of human beings assuming “full control” over themselves through eugenics and pre-natal conditioning. The end would be something like that sought by the transhumanist projects: “We shall have ‘taken the thread of life out of the hand of Clotho’ and be henceforth free to make our species whatever we wish it to be” Lewis writes (2009, 60). (Clotho, a goddess from Greek mythology, and one of the three so-called Fates is a spinner who spins the thread of human life. The length of the spun thread determines how long each person's life will be.)

In this perspective, transhumanism moves beyond modernity and embraces the postmodern. While modernity's exaltation of science sought a new human being based on the old, achieved through a purification by reason, the postmodern deconstruction seeks to make a new human being through the transforming force of the will. One attempts to base the independence of the human person on the self-sufficiency of his all-knowing reason; the other bases it on the autonomy of his will, a will that proposes its own truth. One perceives transhumanism's accommodation of this postmodern concept in its adoption of a fluid anthropology, emptied of any meaningful content. The concept of “human” is given no normative value, dismissed as a construct “without empirical grounding in the findings of science” (Gregory 2012, 19). There is no such thing as a human “project.” Just as postmodernity “seeks to rebuild the human being as an ateleological reality where the outlines of the human evaporate completely” (Pastor and García-Cuadrado 2014, 349), so transhumanism, in dissolving the human specificity, “deconstructs” human beings and attempts to “create” them anew. A parallel may thus be drawn between the transhumanist project and Nietzsche's efforts to “overcome” humanity in pursuit of the “Overman’” (*Übermensch*) (Nietzsche 2006, I [3] 5).

## The Alienation of the Body

The postmodern elimination of the boundaries that define what is “human” begins with the body. As the concrete form and point of limitation of the human person, the body is also “the first element to be replaced in a posthuman ontology” (Valera and Tambone 2014, 359–60). To overcome this limitation, transhumanists propose morphological freedom as a means to self-expression and as “an extension of one's right to one's body” (Sandberg 2013, 56). According to one proponent of the transhumanist cause: “From the right to freedom and the right to one's own body follows that one has a right to modify one's body. If my pursuit of happiness requires a bodily change – be it dying my hair or changing my sex – then my right to freedom requires a right to morphological freedom” (Sandberg 2013, 57).

However, the claim to morphological freedom means that the body ceases to be the irreducible basis of individuality and identity. It does not have an essential relationship to the human subject. Instead, it is extrinsic to the person, arbitrarily related, redundant and replaceable. Accordingly, “if anatomy is no longer a destiny, but the result of a decision that is constantly revocable, the body turns into prosthesis of the Self that is forever in search of an identity. The body is now seen as a sketch, a draft to be corrected” (Russo and Stefano 2014, 459).

Of course, this alienation of the body does not begin with the transhumanist movement. It has deep philosophical roots in rationalist idealism. Cartesian dualism reduced the human person to thought and consciousness, existing as “a sort of inhabitant in a body” (Colombetti 2014, 372) or the eponymous “ghost within a machine.” With the person reduced to mind or rationality, it is logical that, in time, thought itself should be conceived as “a disembodied computable process” (Colombetti 2014, 372) requiring a material substrate, but not necessarily of human flesh. Following this logic, transhumanism envisages novel interactions between human beings and technology: intelligent machines, disembodied consciousness, mind uploading (or downloading). In consequence, the body becomes “a changeable and replaceable substrate; and technology becomes a feature of human ontology” (Colombetti 2014, 372).

Paradoxically, the de-corporealization of the human subject is mirrored by a materialist or mechanistic reduction. In eliminating the complex reality of the human person, transhumanism adopts modernity's materialist conception of the human body as a kind of machine. In this mechanistic view, materialism attempts to explain complex human faculties such as reason, thought, and emotion in terms of chemical processes, which, when adopted by the transhumanist agenda, can be manipulated or augmented to enhance the human organism. As Bostrom writes: “If human beings are constituted by matter that obeys the same laws of physics that operate outside us, then it should in principle be possible to learn to manipulate human nature in the same way that we manipulate external objects” (Bostrom 2005, 3).

However, serious objections are levelled at this materialist reduction that lies at the heart of transhumanist thought. In the first instance, one could sight the objection of Hans Jonas that materialism's reductionist logic cannot fully appreciate living beings. In its dissection of bodies to their constituent parts, it treats them as dead matter. For this reason, Jonas refers to materialism as an “ontology of death” (Jonas 2001, 11). Another objection concerns the reduction of nature to function. If function, and not rational nature, defines the human person, then those members of the human species who lack the physical equipment for function are denied their status as persons, while certain animals (e.g., higher primates) and intelligent machines become potential candidates, thus further confounding human significance and difference (Moltisanti and Solana 2011, 216). Once the concept of the person is determined in terms of functioning rationality, one is rendered incapable of recognizing the intrinsic, ontological dignity of every human being. And once the ontological foundation that makes humans different from every other living being is removed, human beings become merely quantitatively different from other beings (e.g., more complex), and dignity becomes merely subjective (e.g., quality of life, capacity for autonomy etc.) (Moltisanti and Solana 2011, 217). To dignify human beings with an intrinsic worth simply because they are human beings would be akin to a kind of prejudice or speciesism, as professed by the likes of Peter Singer (1993, 55–6; 2009, 572) and his transhumanist protégé, Julian Savulescu (2009, 220).

Without any metaphysical foundation in which to adhere, human nature is thus left devoid of any normative content. A practical consequence of this development is that the idea of “enhancement” is rendered meaningless. Without a normative concept of human nature, enhancement makes little sense and our strivings to improve ourselves are without direction. The fluidity of human nature, and the freedom to create ourselves according to our wishes, renders “any concrete proposal for improvement vague and imprecise” (Murillo 2014, 475). Kass argues: “If … we can no longer look to our previously unalterable human nature for a standard or norm of what is good or better, how will anyone know what constitutes an improvement?” (Kass 2002, 132). For enhancement to have meaning, “[change or] becoming needs Being as its foundation” (Valera 2014, 484). Enhancements require a given structure in which to adhere, in the absence of which we can only speak of change. “Freedom uprooted from nature can no longer aspire to improve nature. It can only aspire to reconfigure it over and over again, as it pleases” (Murillo 2014, 477). And in the end, such reconfiguration of nature is not only without limit, but essentially without meaning and hope as well.

## The Philosophical Limits of Positivism

Transhumanism can therefore be seen as heir to a rationalism that canonizes empirical science and critical reason as the only sources of knowledge about humanity and the world. It thus pins its hopes on the seemingly endless possibilities offered by scientific development. However, this exaggerated confidence in technological reason has drawn wide criticism from diverse quarters. One thing that Christianity and postmodernity have in common is the rejection of an exclusive scientific positivism as making us prisoners of their own methods. By restricting truth to what is scientifically verifiable, reality is rendered smaller and immanent. Ratzinger suggests that one “who can no longer transcend the limits either of his consciousness or of his speech fundamentally can no longer speak of anything at all” (Ratzinger 2009, 80).

The confining of all that can be known to the human knower similarly subverts knowledge. Challenging the absoluteness of positivistic knowledge, Ratzinger draws a distinction between knowledge and understanding, between “*ratio* (reason in relation to the empirical, to the realm of what can be done), and *intellectus* (reason that contemplates the deeper strata of being)” (Ratzinger 2006, 110). He also draws on Heidegger's categories of thought as *calculating* (concerned with making) and thought as *reflective* (concerned with meaning) (Ratzinger 2004, 71).

Knowledge of matter, according to Ratzinger, is intrinsically positivistic, limited to what is measurable and functional. It is, therefore, incredibly useful and valid. However, such knowledge is limited in its scope. It is not concerned with truth as such. It does not “inquire what things are like on their own and *in themselves*, but only whether they will function *for us*” (Ratzinger 2004, 75). Structural knowledge, therefore, cannot penetrate to the essence of reality. As the youthful Ratzinger wrote: “To decipher the physical structure of things is not the same thing as to decode the meaning of existence itself. Rather, it [i.e., meaning] introduces us to the enigmatic character of existence in its full mystery and thus shows us the riddle of our own existence” (Ratzinger 1966, 234).

Metaphysics and faith, on the other hand, are not concerned with the calculable and the positivistic. Contrary to rationalistic prejudice, faith is not provisional knowledge, “a diluted form of natural science, an ancient or medieval preparatory stage that must vanish when the real thing turns up” (Ratzinger 2009, 29). Nor is it some enigmatic system of knowledge, “a colossal edifice of numerous supernatural facts, standing like a curious second order of knowledge alongside the realm of science” (Ratzinger 2009, 30). Rather, Ratzinger defines faith as “an existential attitude, a fundamental decision about the direction of life” (Ratzinger 2009, 35). It is the “disclosure” of a greater reality, open to those who love and trust. It is the acknowledgement that being, including the human person's being, is beyond the measurable and the calculable. In the realm of that which cannot be “laid on the table” and dissected by scrutiny, faith seeks to understand being itself, not knowledge of its products. According to this rationale, Ratzinger writes that “[t]he tool with which man is equipped to deal with the truth of being is not *knowledge* but *understanding*: understanding of the meaning to which he has entrusted himself” (Ratzinger 2004, 77).

Accordingly, the differences between understanding and knowledge do not constitute a contradiction. Ordinarily, faith does not impose on human reason, but rather serves to “awaken” and recall it to itself (Ratzinger 1995, 1). The two come into conflict only when they fail to respect their differences and intrude on each other's domain. However, they need each other. In Ratzinger's analysis, rational discourse is impoverished by the current imbalance between knowledge and understanding, in which everything is reduced to the narrow confines of *ratio*. He suggests that Heidegger was justified in his concern “that in an age in which calculating thought is celebrating the most amazing triumphs man is nevertheless threatened, perhaps more than ever before, by thoughtlessness” (Ratzinger 2004, 71). For all the knowledge gained through technical and scientific thinking, humanity as a whole has not progressed in understanding. By restricting knowledge to what is practicable, human beings risk losing themselves and the reason for their existence. As Ratzinger writes, “man does not live on the bread of practicability alone; he lives as *man* and, precisely in the intrinsically human part of his being, on the word, on love, on meaning” (Ratzinger 2004, 72–3). Love, word, meaning, and other realities like the transcendentals of truth, goodness, and beauty, constitute what is truly human and cannot be reduced to positive categories. To take just one example, Ratzinger highlights the impossibility of the mathematical mind to comprehend the “superfluous wonder” of beauty, its extravagance surpassing explanation by calculation (Ratzinger 2004, 154). In the end, he insists that positivism is incapable of extinguishing belief, that it cannot annihilate the human search for meaning and the yearning for transcendence, because faith, in its seeking for understanding, corresponds to the truth of human nature.

## A Deep Despair

Philosophically restricted to positive categories, transhumanism is incapable of grasping the depths of the human person. While not hesitating in his acknowledgement that, as a scientific method, positivism “is unbelievably useful and absolutely necessary for the mastery of the problems of ever-developing humanity,” Ratzinger insists that “as a philosophy of life [it] is intolerable and the end of humanity” (Ratzinger 2009, 81); that it “is adequate in the technical domain,” but that when it is universalized it “mutilates man” (Ratzinger 2005, 351).

As symptomatic of this “mutilation,” transhumanism radically underestimates the human person's capacity for spiritual transcendence. It is, therefore, incapable of grasping the goods of man, nor of comprehending the sublime object of his hope. Informed by a negative anthropology, it values human life so little that it is willing to gamble away its uniqueness, surrendering humanity to the whims of technology on the chance that it might be enhanced. Whether flowing from callous disregard, unfounded optimism or dark despair, it would seem madness to allow technological power to be wielded by those who possess such a negative conception of human nature. Hans Jonas makes a similar point. In response to the charge of pessimism over his caution of the destructive power of technology, he suggests that “the greater pessimism is on the side of those who consider the given to be so bad or worthless that every gamble for its possible improvement is defensible” (Jonas 1985, 34). In other words, those who consider the human condition so lightly as to risk everything should not be trusted with its future. Transhumanists, with their negative attitude toward the given, should be the last to offer counsel on what is good for the future of humanity. Only those who are truly human, who love and appreciate the giftedness of human life, can be trusted to use technology in a way that fosters integral human development.

## Conclusion

Transhumanism, like other contemporary prospects for human enhancement, offers itself as hope for the future; to “relieve” humanity of its limitations (McKenny 1997, 18) through the application of biotechnology. However, as traced in this paper, the philosophical foundations of transhumanism in scientific positivism render it incapable of understanding the human novelty. In its narrow conception of reality, its limitation of meaning to praxis, and its reduction of the human essence to rationality (mind) or materialism (body), it fundamentally changes our anthropology, strips it of normative value, and distorts the essence of our humanity. Its capacity to know what is good for humanity is, therefore, extremely doubtful.

Furthermore, through the secularization of hope, characterized by an absolute belief in progress through science and technology, the future of humanity is firmly placed within the hands of biotechnology. With unrestrained confidence in the powers of technology, the transhumanist cause is projected as the “new frontier” of exploration—the aspiration and hope for the future. However, from its origins, such optimistic aspirations are rooted in negativity and frustration over humanity's current lot. This sense of dissatisfaction for the present human condition—the sense that we are “not good enough”—is the foundation of the transhumanist dream that opens towards a posthuman future. This façade of hope, therefore, can offer no real reassurance. The transhumanist dream, despite its veneer of hope, is in reality a manifestation of a deep despair.
